# Embedding the rehabilitation treatment specification system (RTSS) into clinical practice: an evaluation of a pilot teaching programme

**DOI:** 10.1186/s12909-022-03861-2

**Published:** 2023-02-02

**Authors:** Jamie Gibson, Jade Sampford, Richard Myers-Ingram, Gareth D. Jones

**Affiliations:** 1 Department of Physiotherapy, Guy’s & St Thomas’ Hospital NHS Foundation Trust, London, UK; 2 Health Education England (HEE), Leeds, UK; 3 Centre for Human & Applied Physiological Sciences (CHAPS), Faculty of Life Sciences & Medicine, King’s College London, London, UK

**Keywords:** Rehabilitation treatment specification system, Clinical reasoning, Physiotherapy, Process assessment, Clinical education

## Abstract

**Background:**

Deficiency in the provision and quality of treatment specification by rehabilitation clinicians impairs the ability to differentiate effective from ineffective elements of treatment. The standardised language of the rehabilitation treatment specification system (RTSS) has been proposed as a countermeasure. To date, there is no evidence of its use in clinical practice and what effect it may have. This study aimed to assess the ability of a pilot teaching programme to embed the RTSS into the clinical practice of an inpatient oncology physiotherapy team. The objectives were to evaluate the teaching programme’s effect on; participants’ familiarity and perceived benefit of the RTSS, its uptake, participants’ clinical reasoning, and their feelings and attitudes towards adopting the RTSS. This study provides an evaluation of the pilot teaching programme which will subsequently inform a larger iteration in an ongoing Health Education England (HEE) project aiming to disseminate and embed the RTSS into physiotherapy practice to improve physiotherapists’ treatment specification.

**Methods:**

A 6-week, multi-modal RTSS pilot teaching programme based upon socio-constructivist theory was delivered to 10 inpatient oncology physiotherapists at a large urban UK trust in 2021. Self-reported measures and clinical case note audits were assessed before and after the RTSS teaching programme to evaluate its effect on RTSS familiarity and perceived benefit, uptake, and clinical reasoning. A post-teaching focus group was undertaken. It was qualitatively analysed using an inductive, independent thematic approach to evaluate clinicians’ reflection and adoption.

**Results:**

Ten participants (8F, 29.4(±3.5) years) with variable clinical experience completed the RTSS teaching programme (six 1-hour lecture/case-based-learning sessions weekly) with 85% mean attendance. Nine yielded complete data for analyses, and 7 participated in the focus group. There was significant improvements in self-reported familiarity and confidence using the RTSS. Furthermore, there was a significant effect of the teaching on self-reported clinical reasoning overall and specifically in knowledge and theory application. But this was not reflected in clinicians’ uptake of RTSS language, nor in the quality of clinical reasoning emergent in their case notes. Qualitative analyses revealed that while clinicians’ conceptual understanding and the relative advantage of using the RTSS in practice was pervasive, they articulated that translating its perceived academic disposition into their clinical practice a challenge.

**Conclusions:**

The RTSS teaching programme was shown to be effective in improving self-reported measures of clinical reasoning, despite clinical uptake of the RTSS remaining low. Future iterations should be tested across physiotherapy specialisms and in a larger sample with consideration of pedagogical and cultural measures to support the clinical diffusion of the RTSS.

**Supplementary Information:**

The online version contains supplementary material available at 10.1186/s12909-022-03861-2.

## Introduction

Absent or heterogeneous specification of rehabilitation treatment makes it difficult to determine the effective or ineffective elements of treatment. This is known as the “black box of rehabilitation” [1]. The Rehabilitation Treatment Specification System (RTSS) is an attempt to mitigate the problem by proposing a set of concepts and a common language that is anticipated to improve the understanding, replicability and clinical transferability of rehabilitation treatments if adopted into practice [2].

## Background

The term “black box of rehabilitation” has been in common use since 1990 [1] and denotes the ongoing struggle to determine what aspects of rehabilitation treatment interventions cause change in patient outcomes. The lack of a shared and standardised language to define and describe rehabilitation treatments serves to perpetuate this phenomenon. The need to resolve this is recognised by the development of multiple reporting guidelines such as the Template for Intervention Description and Replication (TIDieR) [3] and the Consensus on Exercise Reporting Template (CERT) [4]. These guidelines direct researchers towards what information they should describe e.g., the components of an intervention and how to provide specificity to the patient. However, these guidelines do not direct practicing therapists and students towards improving their rehabilitation treatment descriptions not least in challenging the user to propose what and how the described treatment elements cause change in patients. Instead, it is assumed that users will accurately identify this themselves independently of the guidance which means there is a risk that any causal link is simply not considered [5].

The RTSS in contrast provides a conceptual framework and common language to improve rehabilitation treatment description across research, education and clinical practice [6]. The specification framework is embedded in treatment theory [7]. It challenges the clinician to identify a treatment component made up of a singular (measurable) treatment target, patient function that is to be changed directly by the ingredient(s); 1 or more (measurable) ingredients, what the clinician does to modify the target; and mechanism(s) of action (MoA), the causal chain through which the treatment is known or hypothesised to work (i.e. how the ingredients affect the target) [8]. To date, published applications of the RTSS have demonstrated the ability of the RTSS to map targets and ingredients to a patient population or therapy discipline using previous research [9–14]. Yet understanding clinicians’ experiences utilising the RTSS and any effects it has on clinical rehabilitation practice remains to be understood.

One effect hypothesised by proponents of the RTSS is an improvement in clinical reasoning of treatment planning [2]. A clear understanding of what clinical reasoning means or looks like is a challenge because it is a heterogeneous construct with a broad literature base providing a diversity of definitions, theories, and approaches. The ability to assess and develop clinical reasoning is therefore diminished [15]. This is pertinent in the acute rehabilitation of hospitalised patients especially where symptom burden, comorbidities, deconditioning, and other iatrogenic outcomes of signature medical or surgical treatments have significant effects on sensorimotor and cognitive-affective systems - for example inpatient oncology rehabilitation [16]. The framework of the RTSS could provide a more uniformed process for clinicians to explore and articulate their clinical reasoning in treatment planning even in challenging rehabilitation settings because it adopts a common process and language [2]. Utilising the RTSS could therefore engender clinical practice development by conferring clarity in how clinicians clinically reason a treatment approach based on their clinical assessments.

In this study, we designed and implemented a context-specific and theoretically based pilot RTSS teaching programme. The aim was to embed the RTSS into clinical practice by delivering the teaching programme to an inpatient oncology physiotherapy team. Our objectives were to evaluate the teaching programme’s effect on; participants’ familiarity and perceived benefit of the RTSS, its uptake, participants’ clinical reasoning, and their feelings and attitudes towards adopting the RTSS. This study provides an evaluation of the pilot teaching programme which will subsequently inform a larger iteration in an ongoing Health Education England (HEE) project aiming to disseminate and embed the RTSS into physiotherapy practice to improve physiotherapists’ treatment specification.

## Methods

### Study design

The multi-modal teaching programme was implemented into an acute inpatient oncology physiotherapy team over a period of 6 weeks in a large urban UK NHS Trust. A mixed methods approach was used to evaluate the project before and after the implementation of the RTSS teaching programme.

### Description of teaching approach

**Fig. 1:**
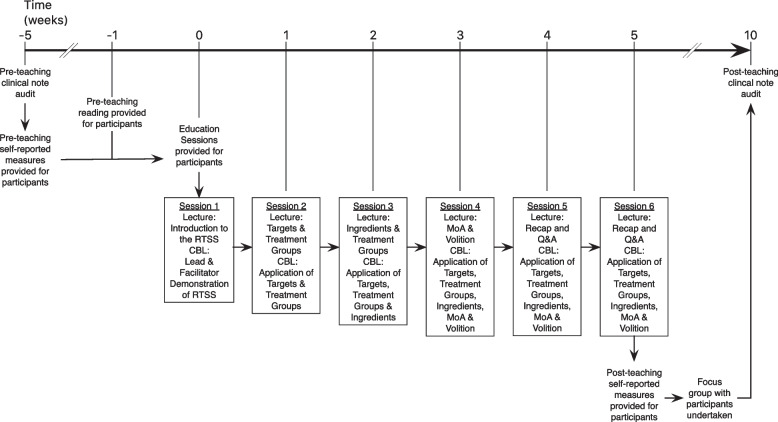
Schematic summarising participants’ path through the project. The RTSS educational sessions lasted for 5 weeks in total (6 sessions; a brief summary of content is provided). Participants were asked to complete self-reported measures at the beginning and at the completion of the education sessions. Clinical notes of the physiotherapy oncology team were assessed for controlled RTSS language approximately 1 month before and 1 month after the education sessions by the research group. Qualitative data were extracted from participants after the completion of the education sessions by the researchers conducting a focus group. CBL – case-based learning; MoA – mechanism of action; Q&A – question and answer sessions; RTSS – rehabilitation treatment specification system

The primary teaching intervention took place face-to-face in a classroom setting in 6, 1-hour sessions delivered weekly incorporating a hybrid approach of lectures and cased based learning (CBL) [17]. The lectures were implemented to cover course aims, introduction of the constructs of the RTSS and interactive applications of the RTSS to clinically relevant case examples. Content of the lectures was informed by the RTSS manual handbook [18], discussion with ACRM’s networking group and from Hart et al’s special communication [6]. The lead and facilitators were all trained via accessing the same resources that informed the course content, as well as participating in two 1 hour sessions where they were given the opportunity to apply the RTSS to different clinical examples created by the lead. Scaffolding [19] of the course content (Fig. 1) was partially guided by the RTSS manual handbook [18], however contrary to the process of specification of the RTSS, the lead and facilitators rationalised that the concepts of Targets and Ingredients should be understood first in order to be able to apply the concepts of MoA and Volition. Course aims were 1. To understand the clinical rationale of the RTSS; 2. To understand the main concepts of the RTSS; and 3. To be able to apply to the RTSS to clinical practice.

At each session, a clinician was expected to present a pre-prepared case to inform the CBL following the lecture component. To standardise structuring of case presentations, clinicians were expected to present the case orally from a prepared written study using a locally accepted standardised format (Additional file 1). After the case presentation, clinicians clarified any outstanding patient details prior to attempting to apply the RTSS. Application of the RTSS was completed using the think-pair-share approach [20] where clinicians were challenged to independently consider targets, ingredients, and the proposed MoA(s) in relation to the treatment planning of the case, prior to pairing-up or joining small groups, and then feeding back their thoughts to the group as a whole. Three facilitators were assigned during the “pair” stage and were instructed to support clinicians on the application of the RTSS to treatment planning. Facilitators did not instruct nor comment on treatment choice because treatment diversity was encouraged to promote group debate. Supplementary to the classroom sessions, clinicians were provided access to an online forum to encourage group discussion as well as an RTSS glossary, manual handbook, and bibliography. Clinicians could also contact course facilitators via e-mail if they preferred.

### Theoretical basis

Physiotherapists require a considered teaching approach because they embody a unique collection of learning needs and barriers [21]. Generally, physiotherapists tend to feel positively towards the adoption of clinical innovations but report a lack of skill in applying them to their practice or hold poor perceptions of the clinical applicability of innovations [22]. Teaching programmes that have proven to be successful in developing application and uptake of physiotherapy innovations have the common characteristic of providing multiple and diverse teaching resources [21]. This supports a multi-modal teaching design approach. In order to combat poor perception of clinical innovations experienced by physiotherapists, the course first focussed on the benefits of the RTSS in clinical practice to highlight it’s “relative advantage” [23]. While perceived relative advantage does not guarantee adoption, it can provide ease of implementation [24].

The use of lectures in education continues to be debated. Some argue that lectures are a passive and dated medium of teaching which often ignores the recipients [25]. Others argue that the efficacy of lectures depend on the content and quality of delivery [26, 27]. Lectures were justified in this programme by their effectiveness in developing engagement and foundational understanding of new complex ideas [28]. In this case, the lectures were used as means of scaffolding [19] to provide a basic understanding of the new language of the RTSS and its application to clinical practice, prior to the clinicians progressing and attempting to use the language in the CBL activity.

CBL is a learning approach whereby a factually based complex problem is used as a catalyst for class discussion, thus creating an active learning role for the student [29]. CBL differs from the problem-based learning (PBL) approach in its requirement of the group to perform advance preparation and the expectation of the facilitators to provide direction to learning points by using guiding questions [30]. The specific context necessary to practice applying the novel RTSS language to the treatment planning of an singular oncology patient case, and ultimately to any case in real-world clinical practice, was provided by deploying a CBL approach. There is evidence of inferior independent learning uptake from CBL in comparison to PBL though. Counterarguments justifying a CBL approach include CBL’s compatibility with lectures [31]; its perceived superior time efficiency, focused learning, and ability to develop clinical skills [30]; and that CBL fosters a collaborative and team-based learning approach to education [32].

The authors predicted that a group of mixed seniority and clinical experience could result in inequality of group contribution and thus opportunity to practice contextual application of the RTSS. To combat this, the think-pair-share approach was introduced. The ‘think’ stage provides an initial phase of silence allowing students time to retrieve knowledge and organise and apply their understanding [33, 34] prior to discussing in the ‘low stakes environment’ of the ‘pair’ stage [35]. This is evidenced to increase engagement and complexity of student response in the group ‘share’ stage [36] thus creating a more equitable opportunity to practice and develop the clinical application of the RTSS.

### Implementation of teaching and data collection

Physiotherapists working with acutely hospitalised oncology patients were selected due to the clinicians routinely encountering complex symptomology and rehabilitation needs with their patients. A retrospective review completed by the recruited team found 92% of patients to have 3 or more co-existing symptoms, and a mean Eastern Cooperative Oncology Group Performance Status (ECOG PS) [37] of 2.1 [38]. This complexity was hypothesised to provide a rich and challenging context for treatment planning and thus provide a rigorous test of the RTSS teaching programme.

The team consisted of 10 rotational and static physiotherapy staff employed at various pay-grades (Agenda for Change (AFC) bandings 4–8 [39]). We deliberately selected a group with diverse job descriptions as an opportunity to assess the generalisability of RTSS across the physiotherapy workforce spectrum. Physiotherapy students were however excluded because of the high likelihood there would be missing data due to inflexible placement length. Formative preparatory material was provided (a reading list and a prepared bespoke slide presentation to introduce the concept and nomenclature of the RTSS) to all participants simultaneously at least 1 week before the RTSS teaching programme commenced (Fig. 1). The evaluation of the programme utilised the Grey Box approach of Scriven’s three boxes model [40, 41] because it endorses measurement of both the efficacy of the teaching design, and internal mechanisms that modulate its efficacy.

### Measures and data analysis

All measures were completed prior to the release of the pre-reading and repeated at a maximum of 4 weeks after completion of the RTSS training programme to enable before-after changes to be assessed (Fig. 1). The measures adopted assessed change across different constructs collectively designed to determine how well the RTSS was embedded in clinical practice; familiarity and perceived benefit of the RTSS, its uptake, its effect on clinical reasoning, and a retrospective reflection of clinicians’ feelings and attitudes towards the training programme and adopting the RTSS.

### Familiarity & perceived benefit

A self-reported questionnaire was used to determine clinicians’ familiarity and perceived benefit of the RTSS. It was deployed before and after RTSS training. The questionnaire consisted of 11 questions in total; 8 statements for which participants were instructed to respond using a 5-point Likert scale (1, strongly disagree; to 5, strongly agree). Two further dichotomous (yes/no) statements were used to assess participants’ familiarity and use of the RTSS, and a final numerical rating scale (1–10) was used to assess how clinically beneficial the participants found the RTSS (Additional file 2). Changes in completed paired questionnaire responses before and after RTSS training was assessed using Wilcoxon signed-rank tests independently for each question.

### Uptake

A frequency of controlled vocabulary audit was carried out on patients’ physiotherapy clinical case notes (documented using the SOAP format as per professional guidance [42, 43]). Notes for patients where an initial assessment was completed were sequentially selected during March–April 2021 (pre RTSS training, *n* = 18), and June–July 2021 (post RTSS training, *n *= 18). Patients initially assessed for whom no physiotherapy treatment was indicated were excluded. Fourteen controlled vocabulary words or terms were adopted from the glossary of the manual handbook for the RTSS [18] (Additional file 3).

Cumulative incidence of words/terms within individual note entries was rated by one of 3 authors (JG, RMI, JS). Inter-rater agreement was determined a priori using two-way random effects model intraclass correlation coefficients ICC_(2,1)_ for absolute agreement across the raters for total-count terminology incidence overall, and for each controlled vocabulary item in 15 clinical case notes sequentially selected and adjusted to include words/terms by an independent author blinded to the rating procedure (GDJ). The strength of agreement for total vocabulary count (ICC_(2,1)_ = 0.724 (95%CI 0.544 to 0.888)) was interpreted as good based on published guidelines [44, 45] (Additional file 4). The effect of training on total word count (from 14 words/terms) across 18 notes screened before and after RTSS training was assessed by using an independent samples student’s *t*-test.

### Clinical reasoning

The Self-Assessment of Clinical Reasoning & Reflection tool (SACRR) [46] was selected as the non-work-based assessment of clinical reasoning. The SACRR is a 26-item questionnaire that describes the cognitive processes of clinical reasoning where respondents self-rate descriptions on 5-point Likert scale. The tool is designed to measure 4 constructs of clinical reasoning (Knowledge and theory application; Decision making; Dealing with uncertainty; and Self-reflection/reasoning) [47]. Changes in self-reported clinical reasoning behaviour between pre and post RTSS training was determined by assessing the median difference in SACRR total scores, and the median difference in each of the 4 SACRR domain sub-scores using exact sign tests. To date the SACRR has been used in undergraduate education, its reliability and validity in assessing clinical reasoning has been adequately reported [48–51]. To provide a real-word measurement of practicing clinician’s clinical reasoning, a work-based assessment was sought.

A work-based assessment of clinical reasoning within the same clinicians’ SOAP documentation as assessed for uptake was undertaken using a rubric. Rubrics have demonstrated ease of use, reliability, and validity in measurement of physician’s clinical reasoning [52]. To the authors’ knowledge, there was no precedent rubric available to assess clinicians’ notes. One was therefore designed by consensus of 4 senior rehabilitation physiotherapists (Additional file 5). The rubric provides criteria of scoring low, moderate, and high quality for 3 clinical reasoning components; problem list formation, treatment goals, and treatment planning.

Inter-rater agreement across 3 raters was determined a priori using Fleiss’s Kappa statistics for each of the reasoning components. Raters independently rated the same 15 clinical cases used to determine the reliability of word/terms frequency uptake of the RTSS. There was moderate agreement between rater judgments for the problem list formation [κ(95%CI) = 0.538 (0.530 to 0.545); *p* < 0.001], the treatment goal reasoning [κ(95%CI) = 0.527 (0.520 to 0.534); *p* < 0.001], and the treatment planning rubric components [κ(95%CI) = 0.466 (0.459 to 0.473); *p* =0.001] (Table 1).

**Table 1:** Summary of inter-rater agreement using the clinical reasoning rubric. Data are shown for overall agreement and for each rating of quality and are based on 3 independent raters of 15 clinical cases

Clinical Reasoning Component	Inter-Rater Agreement				
	Overall κ	(95%CI)	Quality		
			High κ	Moderate κ	Low κ
Problem list formation	0.538	(0.530 to 0.545)	0.515	0.554	0.550
Treatment goal reasoning	0.527	(0.520 to 0.534)	0.659	0.177	0.544
Treatment planning	0.466	(0.459 to 0.473)	0.527	0.306	0.544

κ - Fleiss’s Kappa, CI - confidence interval

Differences in the multinominal probability distributions before and after RTSS training between the proportions of rated quality using the rubric was assessed using Fisher’s exact tests [53] for each of clinical reasoning components. In the cases where there was a significant difference across quality, post-hoc pairwise comparisons were made using multiple z-tests of two proportions with a Bonferroni adjustment where statistical significance was accepted if *p* < 0.0167. For all quantitative statistical tests (SPSS v26, IBM Corp, Armonk, NY, USA), significance was assumed if *p* ≤0.05.

### Adoption & reflection

Upon completion of the programme, all therapists were invited to take part in a semi structured focus group to provide insight into clinician’s perspectives on the adoption of the RTSS in clinical practice, and the efficacy of the teaching design (Additional file 6). Structuring of the focus group questions was directed by reflection and feedback of the course lead and facilitators alongside the clinicians’ responses to the post-teaching questionnaire. The focus group duration was 52 minutes, led by one of the authors (JG) with another author acting as an assistant moderator (JS). Audio responses were recorded and transcribed verbatim. Physical response observations were captured from the assistant moderator’s field notes.

Focus group data were analysed by the lead and assistant moderator. The lead moderator read each transcript in full using an inductive thematic analysis approach [54] to identify main themes and related sub-themes emerging from participant perspectives. Colour coding was used to organise emerging themes which were summarised in proprietary software (Microsoft Excel, Microsoft Corporation, Redmond, WA, USA). To ensure credibility of the analysis, the focus group was also analysed by the assistant moderator. Data were cross-referenced and duplicates removed. The lead and assistant moderator compared and reached consensus agreement on the final set of themes which were then drafted with supporting quotations.

## Results

### Participants

Ten physiotherapists (8 females, 2 males; mean (±SD) age 29.4 (±3.5) years (range 25–35)) working for 4 (±2.7) years (range 9 months-9 years) participated. Mean attendance at each (*n* = 6) scheduled training sessions was 85% (±6%), range 75–92%. Pre and post RTSS training data was available for analyses in 9 matched pairs by grade; 2 were employed contractually at AFC [39] Band4, 1 Band5, 2 Band6, 3 Band7, and 1 Band8a (higher numbers represent higher pay-band).

### Familiarity & perceived benefit

Nine participants returned questionnaire data (11 questions) both before and after RTSS training. Missing data meant question 9 (Q9) was based on 8 pairs, Q10 on 7 pairs, and Q11 on 4 pairs. While there were positive changes on some of the Likert scales (Q1 and Q2 (median change 4 to 5), Q7 (3 to 4)), and in the numerical rating of the RTSS (Q11 (3 to 7.5)), none were statistically significant. There was no change in median scores on Likert scales for Qs 3–6 and Q8. In contrast, there were statistically significant median positive change in Q9 (familiarity of the RTSS) where 5 participants changed their responses positively and 3 remained the same (median difference of 1 point [*z* = − 2.236, *p* =0.025]), and in Q10 (use of the RTSS in practice) where 5 participants changed their responses positively and 2 remained the same (median difference of 1 point [*z* = − 2.236, *p* =0.025]).

### Uptake

There was no significant effect of RTSS training on the total frequency counts of 14 controlled vocabulary words/terms audited across 18 clinical cases pre and post RTSS training. There were 4 words/terms counted in total pre-RTSS training (a mean (±SD) of 0.220 (±0.548) words/terms per case), and 7 post-RTSS training (a mean of 0.390 (±0.698) words), a mean (95%CI) increase of 0.167 (− 0.258 to 0.592) words per case [*t* (34) = 0.797, *p* =0.431] (Table 2).

**Table 2:** Total counts of controlled vocabulary words/terms assessed across 18 independent clinical case notes before and after RTSS training

No.	RTSS Controlled Vocabulary Word/Term	Total Count Across 18 Case Notes	
		Pre-RTSS Training	Post-RTSS Training
1	Rehabilitation treatment specification system or RTSS	0	0
2	Specify/Specified/Specification	0	0
3	Treatment component	0	0
4	Aim	1	7
5	Target	0	0
6	Ingredient	0	0
7	Mechanism of Action	0	0
8	Treatment group	0	0
9	Organ/Organ function/Organ system	0	0
10	Skill (including Activity-like or Function-like) and/or Habit	0	0
11	Representation	0	0
12	Dose/Dosage/Dosing/Dosing parameters	0	0
13	Progression	3	0
14	Volition/Non-volition	0	0
	Total	4	7

### Clinical reasoning

There was a significant median (IQR) increase in overall SACRR scores of 8 (5–12) points after RTSS training [*p* =0.004]. For the SACRR Knowledge and Theory Application domain, 8 participants increased and 1 reduced their scores. There was a significant median increase of 4 (2–6) points [*p* =0.039]. For the SACRR Decision Making domain, 5 participants increased, 1 reduced, and 3 saw no change in their scores. While there was a median increase of 3 (0–6) points, it was not statistically significant [*p* =0.219]. Similarly, for the SACRR Self-Reflection/Reasoning domain, 7 participants increased, and 2 reduced, their scores and there was a median increase of 1 (0–5) points which was not statistically significant either [*p* =0.180]. Finally, for the SACRR Dealing with Uncertainty domain, 4 participants increased, 1 reduced, and 4 saw no change in their scores and there was non-statistically significant median difference of 0 (0–2) points after RTSS training [*p* =0.375] (Table 3).

**Table 3:** Median (IQR) data for the Self-Assessment of Clinical Reasoning & Reflection (SACRR) tool

SACRR Domain	Max. Score	RTSS Teaching Programme		Median Difference	
		Before	After		Sig
All	130	94 (90–99)	104 (99–109)	8 (5–12)	**
Knowledge & Theory Application	25	15 (14–17)	20 (18–22)	4 (2–6)	*
Decision Making	50	38 (35–38)	40 (38–41)	3 (0–6)	ns
Self-Reflection/Reasoning	20	15 (14–16)	16 (16–19)	1 (0–5)	ns
Dealing with Uncertainty	35	27 (24–29)	29 (26–29)	0 (0–2)	ns

*IQR* interquartile range values, ns not statistically significant, Sig-statistical significance; ** represents statistical significance at the *p* ≤0.01 level; * represents statistical significance at the *p* ≤0.05 level

While there was no significant difference in the distributions of rating quality according to the clinical reasoning rubric in problem identification [*p* =0.783] and treatment goals [*p* =0.486] components, there was a significant difference in the treatment plan component [*p* =0.041]. Post-hoc comparisons revealed a statistically significant reduction in the proportions of low-quality clinical reasoning (94.4% pre and 61.1% post RTSS training, *p* =0.016) and a commensurate significant increase in moderate quality clinical reasoning within this component (5.6% pre and 38.9% post RTSS training, *p* =0.016; Table 4).

**Table 4:** Count (proportions) of quality ratings based on the predetermined rubric from 18 sets of clinical notes pre and post RTSS training

	Clinical Reasoning Component								
	Problem List Formation			Treatment Goal Reasoning			Treatment Planning		
Quality Rating	Pre	Post	sig	Pre	Post	sig	Pre	Post	sig
High	0 (0%)	0 (0%)	ns	0 (0%)	1 (5.6%)	ns	0 (0%)	0 (0%)	*
Moderate	9 (50%)	11 (61.1%)		0 (0%)	1 (5.6%)		1 (5.6%)	7 (38.9%)	
Low	9 (50%)	7 (38.9%)		18 (100%)	16 (88.9%)		17 (94.4%)	11 (61.1%)	
Total	18 (100%)	18 (100%)	–	18 (100%)	18 (100%)		18 (100%)	18 (100%)	–

sig - statistical significance across ratings, ns - not significant

* represents statistical significance at the *p*≤ 0.05 level

### Adoption & reflection

Seven clinicians [mean(±SD) age 28.3(±3.4) years, Band range 4–7, working for 3.3(±1.9) years] participated in the focus group which was 52 minutes in duration. Inductive independent thematic analysis resulted in the following 4 qualitative themes describing the clinicians’ understanding of the concept of the RTSS, perceived relative advantage of the RTSS, experiences of the teaching design, and seniority and expectations that influenced the clinical adoption of the RTSS. Themes are explained with illustrative quotes.

### Theme 1: perceived understanding of concept

- Clinicians outlined a broad understanding of the RTSS following the teaching programme, including the definitions of its novel concepts.
  i. *I think we all understand the concept of volition, target, aim* etc.
  *(Participant 3, band 5)*
- Clinicians were also able to outline the conceptual value of the RTSS and how it could change their clinical practice. In particular the act of specifying the different concepts seemed to enable the application of theory and knowledge as well as help clinicians challenge and reason their treatment choices. The clinicians perceived this as a benefit due to the outcome of more appropriate treatment plans.
  i. *It kind of forces you to think: oh but why am I doing that? What am I aiming to achieve? What system am I trying to influence? And actually that just kinds of takes you back to those things we do but we just do quite automatically, and sometimes by thinking more about it you realise oh maybe that wasn’t the most appropriate plan or I could have been more specific.*
  *(Participant 7, band 7)*
- Despite understanding the terminology, clinicians reported a lack of confidence in applying the RTSS to their own clinical practice which ultimately led to non-use. Overall, clinicians felt that they needed more practice in applying the RTSS outside of a clinical setting prior to using it in clinical practice. A significant barrier to clinical use was applying the RTSS to patient documentation, again clinicians desired training outside of the clinical setting.
  i. *I think I’ve got a basic understanding of what the RTSS is and how it can be used in clinical practice, I feel that, I don’t feel like I can transfer that to every patient case, I don’t feel confident doing it in routine practice….yet.*
  *(Participant 7, band 7)*
  ii. *I personally feel like I need more practice (documenting) in a safe environment.* *Versus* *writing it in a formal documentation on the ward for a patient that everyone can see and look at, I don’t feel like I’m confident to do that yet, feel like I want to practice more before I feel like I can start doing that right now.*
  *(Participant 7, band 7)*

### Theme 2: perceived relative advantage of the RTSS

- Clinicians reflected on their structuring of rehabilitative treatment planning alluding to a tendency of trying to treat everything at the same time. The treatment component approach of the RTSS provided a countermeasure to help clinicians breakdown treatment into manageable targets.
  i. *Sometimes you feel like you don’t have the structure and you’re just trying to achieve with the approach of trying to hit everything at the same time, it helped me think okay you don’t have to try and tackle everything, just focus on this one bit*
  *(Participant 1, Band 4)*
- A 7-day working pattern highlighted the importance of communicating treatment plans and targets effectively, clinicians were able to identify how using the RTSS to specify treatment components could lead to easier identification and continuity of treatment plans. Shortcomings of the current approach to documenting treatment were also reflected upon, particularly the difficulty in understanding the direction of treatment.
  i. *I think as well, if you were doing 7 day working, how actually it could make it easier for the next physio that comes along that may not know the patient or be aware of it, to pick up what you were thinking, like, in, referring to the RTSS, what were you thinking the issues were here, what are you aiming for? What is your target at the end of it? How are you going to go about that? Like, it’s a bit more structured rather than just writing AROM exercises, it’s more specific. It’s a lot easier then I suppose for continuity of what everyone’s aiming for.*
  *(Participant 4, Band 6)*
- Clinicians explored their relationship with specifying volitional targets and ingredients. Although identifying volition as a driving factor for treatment outcome, clinicians felt that it was often underspecified leading to a feeling that it was not prioritised. Clinicians appreciated how the RTSS brings volition to the forefront of treatment planning.
  i. *I think by also having a section where you think about …other influences outside of, the bigger picture of motivation… and volitional. Is definitely something that I think is important, because I think, it can be easily undermined, when actually it is such a driving factor of our therapy, if there’s no engagement we can only do so much.*
  *(Participant 6, Band 6)*
- Clinicians highlighted the complexity of clinical reasoning as a concept and how they can find it difficult to teach to students and clinicians. The RTSS was seen as a tool to help break down clinical reasoning into a tangible process that could be more easily taught.
  i. *I can see it being a useful model to kind of teach to students or like juniors like myself. Just trying to teach how clinical reasoning works, it kind of is a vague concept to explain to maybe a student, but I think it kind of breaks it down quite a systematic way, so if you taught it in Uni, a student would be like: Yeah, okay I get what clinical reasoning is now.*
  *(Participant 3, Band 5)*
- Clinicians believed that being more explicit with the clinical rationale of the RTSS during the teaching programme could have supported its uptake. References were also made to the fact that there is little supporting literature evidence on the efficacy of the RTSS in clinical practice.
  i. *One thing…how to support the effectiveness of it, and make more of a convincing case for it. That would be nice.*
  *(Participant 3, Band 5)*
- Despite the teaching programme’s ability to demonstrate MoAs as a construct that could improve application of theory and evidence to practice, the process of specifying the MoA was ultimately perceived by the clinician as an academic concept and they questioned its relevance in routine clinical practice.
  i. *Like I can’t think of a time where I couldn’t say I necessarily applied it clinically, cause’ I think, I think its…. Like it’s quite academic, I understand why it’s definitely good to think about, but, I can’t think of a time where with a patient I would either, document MOA more so than I did prior to taking the course..*
  *(Participant 3, Band 5)*

### Theme 3: experience of teaching design

- Clinicians reported improved retention of RTSS language via the opportunity to apply the language from the lecture within CBL, thus supporting it as a method of practicing the skill of utilising the RTSS. However, clinicians experienced difficulty when simultaneously attempting to apply the RTSS and understand a complex case presentation. Clinicians stated they’d prefer to apply the RTSS to cases of lower complexity to begin with.
  i. *I think retention of information is easier when there is application to it as well. I feel like the language was one thing but actually when it came to case studies if you were actually able to use that language into an actual example it was retained better.*
  *(Participant 6, Band 6)*
  ii. *I think we very much do bring like you said the most complex patients, and actually, that’s not to say that you can’t apply those to the model but when you’re still trying to grasp the concept and the language and everything, the most complex patients are probably the hardest ones to do that*
  *(Participant 7, Band 7)*
- The use of small group working and inclusion of staff of all experiences within CBL was felt to improve engagement and provide diversity of feedback, enriching the level of clinical reasoning and treatment discussion. Some clinicians were concerned that the direct transition from lectures to small groups may have resulted in errorful learning. Clinicians highlighted a desire to be “expert learners” prior to practicing with their peers to prevent this. The presence of a facilitator during small group working was seen as a helpful countermeasure to prevent errorful learning.
  i. *You just got a wider range of point of views and experience I think, like having, like a (band) 7,8,6,5 or a 4 it’s just a lot, loads of difference experience coming together. And loads of different ideas and ways of clinically reasoning which, I think was highlighted in the 2nd to last session or last session where a couple of us were aiming for the same target but we had just gone about it in a different way.*
  *(Participant 4, Band 6)*
  ii. *And then, cause’ we went immediately to a small group, so not all of us were expert, sometimes I think, it was the blind leading the blind. So accidentally I was learning false truths.*
  *(Participant 6, Band 6)*
  iii. *I think it was good to be in smaller groups, but it was a real benefit when we had some facilitating like when you kind of came and joined the group and guided so that we avoided what they (participant 4, Band 6) was just talking about, and then, kind of, if we said something that was perhaps off the beat and track he would kind of guide us back.*
  *(Participant 5, Band 6)*
- Although not a formal part of the teaching design, one clinician highlighted the benefits of the opportunity to see a patient with the facilitator. The “real time” use of the RTSS demonstrated how the framework could be applied in a clinical setting contributing to improved understanding beyond classroom teaching approaches.
  i. *I think a turning point with me was when I saw a patient with (facilitator) and then he actually used it in real time with a patient, I was like oh, now I kind of understand it a bit more, than I think I did when just doing the theory. Actually, having a real example of a patient, what he would, where he would put it and him explaining it to me.*
  *(Participant 4, Band 6)*
- A consensus was reached among clinicians that the RTSS presented a significant change to practice and a course length of 6 weeks was insufficient to implement this change.
  i. *I think that’s hard to do in 6 weeks (group consensus: 1 member: Yeah). Change you, the way you do, change the way you’ve always done it, it’s always going to be tricky to do in 6 weeks.*
  *(Participant 1, Band 4)*

### Theme 4: seniority and expectations

- Senior clinicians identified their own role in supporting a culture conducive to improving uptake amongst their team members. But a lack of confidence and competence in knowing how to support their team members to implement the RTSS both during and following the teaching programme contributed to them ultimately not encouraging use of the RTSS. Tailored support for senior staff was identified as a countermeasure.
  i. *A lot of what they felt was that their confidence in knowing how to support the team with it in actually day to day clinical, was lacking, so it wasn’t something we were necessarily reinforcing or driving as part of team ethos in the rest of the week, which, potentially could have helped with that, is something we thought.*
  *(Participant 7, Band 7)*
- Clarity around the future aspirations of the teaching programme contributed to the senior clinician’s attitudes and behaviours towards the RTSS. They expressed wanting to know whether the teaching programme was a temporary project or whether this was part of a longer term department directive.
  i. *The aim of us having this teaching this work done is that this is how we then approach our patient care, is that the expectation in…a year, 2 years everyone within GSTT physio approaches their patient rehab in this way.*
  *(Participant 7, Band 7)*

## Discussion

Our aim in this pilot study was to embed the RTSS into clinical practice by designing and implementing a novel, yet theoretically based RTSS teaching programme to a team of inpatient oncology physiotherapists. Our objectives were to evaluate the teaching programme’s effect on; participants’ familiarity and perceived benefit of the RTSS, its uptake, participants’ clinical reasoning, and their feelings and attitudes towards adopting the RTSS. We are to our knowledge the first group to attempt to do this. The main findings were that while the teaching programme was feasible to implement, was accepted by physiotherapists of all experiences, and participants recognised the potential advantages of the RTSS; its uptake in clinical practice remained low following the teaching programme. We contend that physiotherapists’ clinical reasoning may be developed if the language of the RTSS is adopted in clinical practice. We observed that there were improvements following RTSS teaching in self-assessed clinical reasoning overall, in the knowledge and theory application domain. Yet RTSS uptake remained poor despite the teaching and there was no change in other clinical reasoning domains, nor in the quality of clinical reasoning emergent in case notes, presumably because the diffusion of clinical innovation is complex, takes sufficient time, and is contextually specific [24]. Critically, the focus group data provides an insight into the properties of the teaching programme that may have affected clinical uptake.

A socio-constructivist approach guided the design of this pilot programme in particular the use of lectures and CBL as scaffolding strategies. This theoretical underpinning resonated with staff as evident in qualitative statements (e.g. focus group comment 3.a.i.). However, other clinicians struggled with time, both within sessions and the course overall. A 6-week duration was found to be too short, and clinicians also wished for more time dedicated to both the lectures and CBL. Given that current resources are likely to remain similar in the next iteration of this programme, this challenges the project designers to consider what other methods could support the clinician’s understanding and application of the RTSS.

Pre-teaching could be one solution to developing understanding of the RTSS. Upon exploration, clinicians agreed on a preference of interactive resources as opposed to the bibliography or handbook which were provided as means of pre-teaching. Within the project’s available resources, what could provide widespread interactive pre-teaching? One possible solution could be e-learning. A considered e-learning module can provide the benefits of widespread and interactive delivery of teaching requiring minimal physical resources such as space and teachers. Additionally it could provide the means of remote and widespread assessment and the opportunity to provide sustained and instant feedback which can be beneficial to learning outcomes [55].

The programme did not explicitly instruct clinicians on how or when to apply the RTSS to documentation to observe its natural development within notes. This approach resulted in low confidence in applying the RTSS to patient documentation across all clinical bandings. Low confidence can serve as a predictor for reduced competence [56] making the identification of maladaptive variables affecting confidence imperative. This approach also overlooked the role of the written language of the RTSS as another semiotic mediator on clinicians’ behaviour [19]. By not providing direction for documentation, it is possible that we denied participants a tool that could help to internalise the clinical meaning of the language and what it means in context to clinical practice. Future iterations of the programme should incorporate formal teaching on how to implement the RTSS into clinical documentation.

Interestingly, clinicians identified group dynamics as another mediator of confidence. Senior staff felt a lack of confidence in supporting junior staff when attempting to apply the RTSS and junior staff felt that without senior facilitation, they were learning “false truths” from their peers. Vygotsky proposes that learning is socially mediated, and beliefs is one such semiotic mediator that forms an individual’s higher mental functioning within society [19]. This finding is evident in healthcare with diffusion of clinical innovations being influenced by the values and norms of the leader of the group [24] as well as their attitude towards an innovation [57]. Thus, by neglecting the confidence of senior staff, it is possible we obstructed the adoption of the RTSS as a cultural norm, thereby limiting the facilitation of its uptake amongst the more junior members. Recognising their influence, methods to support senior staff should be considered during the delivery of the teaching programme, but also beyond the teaching programme to promote the longer-term development of the RTSS as a cultural norm.

The perceived relative advantage of an innovation can determine its diffusion [23, 57]. Our data demonstrated an awareness of the perceived relative advantage of the RTSS by clinicians identifying the ability of the RTSS to improve problem list generation, problem solving, theory application, and improve continuity of treatment. Paradoxically, clinicians also felt the lack of prior evidence supporting the RTSS’s clinical efficacy in comparison to current practice presented a barrier to its uptake – a position in keeping with clinical innovation adoption being dependent on the depth and quality of supporting evidence [58]. Indeed, if clinical adoption of the RTSS is dependent on the strength of an RTSS evidence base, then it is perplexing that attempts to provide evidence of the RTSS influencing clinical outcomes is constrained by poor RTSS clinical adoption. If anything, this paradox supports investing in future iterations of this project. This is because the project aims to implement the RTSS in practice on a wider scale and thus provide evidence of what effect it has on clinical outcomes, and expose more clinicians to its clinical reasoning advantages and clinical outcome benefits.

We must also consider the contribution of health concepts to the uptake of the RTSS. Health concepts are an individual’s view of health care and therapy that influence how they interpret and act upon knowledge. Given that clinicians are individuals with views, health concepts can influence clinicians’ professional practice [59]. The framework of the RTSS presents a new health concept for clinicians. Those who choose to engage with it are subsequently tasked with changing their treatment planning behaviour. This is not a trivial challenge because a clinician’s treatment planning behaviour has been a process developed and informed by years of pre-registration study and clinical practice. For example, if a clinician’s treatment planning has been informed to date by a predominantly *enablement* theory approach (predicting treatment-induced effects on more distal aspects of function [60]), it is unrealistic to expect them to seamlessly incorporate a *treatment* theory approach (predicting the direct effects of rehabilitation treatments [60]) incumbent on adopting the RTSS in clinical practice. If physiotherapist health concepts are formed from pre-registration study and continue to be shaped within clinical practice, sustained uptake of the RTSS will be dependent on its larger scale implementation within post, and pre-registration practice.

### Study limitations

The interpretation of our statistical hypothesis tests and the generalisability of our findings should be made with caution given the modest sample size in this preliminary study. We acknowledge that while the complex rehabilitation needs of oncology inpatients provided a robust environment to apply our RTSS teaching programme, its applicability to other physiotherapy specialisms is yet to be understood. We plan to incorporate future iterations of the programme to a spectrum of clinical teams with a larger sample size of participants. Definitions and approaches to understanding clinical reasoning vary widely [15] resulting in difficulty in identifying the most appropriate measures of assessment. The measures selected in this project aimed to provide the content authenticity and validity of work-based assessments and the broad sampling ability and control of non-work-based assessments [61]. We acknowledge that having a single rater undertaking all case-note assessments of RTSS uptake and clinical reasoning would be preferable to 3 raters. Our a-priori assessment of the 3 raters’ agreement revealing good and moderate agreement among them provides adequate mitigation though. Even so, the novel clinical reasoning rubric remains unvalidated and its psychometric properties are yet to be assessed. Resource limitations meant a pre-post design was selected. Given that even high quality evidence is traditionally slow to translate into physiotherapy practice [62] and the mediating effects of clinician confidence and senior support likely requiring support beyond the teaching programme, future iterations will include a sufficient follow up period to assess for sustained changes.

## Conclusions

This multi-modal teaching programme pilot was accepted by staff and resulted in self-reported clinical reasoning improvements. Despite this, our quantitative results showed that the teaching design failed to encourage meaningful uptake of the RTSS into clinical practice. We have presented a discussion of why this might be, based on the combined quantitative and qualitative results, and how the programme could be progressively improved by extending the teaching period beyond 6-weeks, introducing pre-teaching using e-learning approaches, incorporating *how* to document RTSS language into clinical case-notes, and designing the teaching to influence clinical leaders. Within its current resources, future iterations of the teaching programme should consider socio-cultural influences and practical changes to the teaching design to support the diffusion of the RTSS. Application to a larger population across multiple specialisms as well as a longer follow up period will also help to provide a more accurate understanding of the efficacy of the teaching programme in supporting the sustained adoption of the RTSS.

## Supplementary Information

**Additional file 1.** Preparatory document for case study sessions used to inform and standardise case history and treatment planning presentation.

**Additional file 2.** Questionnaire provided to clinicians pre and post RTSS teaching programme to measure familiarity and perceived benefit of the RTSS.

**Additional file 3.** Outline of terminology included in frequency of controlled vocabulary audit completed at pre and post RTSS teaching Programme.

**Additional file 4.** Inter-rater reliability data for the Frequency of Terminology Vocabulary Audit completed at pre and post RTSS teaching Programme.

**Additional file 5.** Clinical Reasoning Rubric of Documentation Assessment used to measure quality of clinical reasoning in SOAP documentation, complete at pre and post RTSS teaching programme.

**Additional file 6.** Script that guided semi structured focus group used to measure adoption and reflection of the RTSS teaching Programme.

## Data Availability

The datasets used and/or analysed during the current study are available from the corresponding author on reasonable request.

## Abbreviations

RTSS: Rehabilitation Treatment Specification System
ACRM: American Congress of Rehabilitation Medicine
TiDieR: Template for Intervention Description and Replication
CERT: Consensus on Exercise Reporting Template
MoA: Mechanism of Action
HEE: Health Education England
CBL: Cased-Based Learning
PBL: Problem-Based Learning
AFC: Agenda For Change
ECOG PF: Eastern Cooperative Oncology Group Performance Status
SPSS: Statistical Package for the Social Sciences
ICC: Intraclass correlation
SACRR: Self-Assessment for Clinical Reasoning and Reflection
SOAP: Subjective, Objective, Analysis and Plan
SD: Standard Deviation
IQR: Interquartile Range
