# Supplementary material for: Embedding the rehabilitation treatment specification system (RTSS) into clinical practice: an evaluation of a pilot teaching programme
Source: BMC Med Educ. 2023 Feb 2;23:85. doi: 10.1186/s12909-022-03861-2 (PMC9896736; doi:10.1186/s12909-022-03861-2)
Supplement: Supplementary file 1 — Additional file 1. Preparatory document for case study sessions used to inform and standardise case history and treatment planning presentation. [file 12909_2022_3861_MOESM1_ESM.docx]

Treatment Planning Sessions Utilising PPIF, the RTSS, & COM-B

Table S1: PPIFF (Pathology-Prognosis/Individual/Function-Fitness) & RTSS (Rehabilitation Treatment Specifiaction System) Approach for Clinically Reasoned Case Discussion Session in Physiotherapy.
COM-B – capability, opportunity, motivation behavioural model [1]; DOB – date of birth; FITT – frequency / intensity / time / type [2]; LOS – length of stay; PA – physical activity

| **Patient Hospital No:** | **DOB/Age:** |
| --- | --- |
| **Sex:** | **Admission Date/LOS:** |
| **Resident Borough/Town/City:** | |
|  | |
| **Pathology-Prognosis:** (Clinical Diagnosis(es), Rate of change, Predicted Disease/Treatment Trajectory, Predicted Rehabilitation Approach e.g. Preventive or Maintenance / Restorative / Supportive or Adaptive / Palliative ) | |
|  | |
| **Individual:** (Factors Influencing Rehab: COM-B [1] / Mood / Cognition / Self-Efficacy / Social Support / Social Environment / Socioeconomics / Community Integration) | |
|  | |
| **Function:** (Premorbid Functional level / Current / Potential / Movement Systems Assessment & Measures) | |
|  | |
| **Fitness:** (Premorbid & Current PA /Exercise / Expectations / Endurance / Ex tolerance / Dosing - FITT principles [2]) | |
|  | |
|  | |
|  | |

Expectations of Treatment Planning Session

1. The number of patients to be discussed per session will be predetermined to plan timings.
2. You should be able to present a brief, spoken summary of the patient you are bringing to the case discussion and treatment planning session. Aim to present PPIFF per patient in ≤15mins
3. The PPIFF summary can include relevant presenting condition, history of presenting condition, length of stay, past medical history and significant medical interventions to provide context
4. You should aim to discuss the PPIFF as a summary to provide the context for any challenges you are facing and why. You should be able to make suggestions on how their rehabilitation and discharge planning is likely to progress in light of the PIFF information you have.
5. It is expected that you will have specified potential treatment targets and ingredients using the RTSS format and that these will encourage discussion within the session to help you formulate and specify a reasoned and justifiable treatment plan.
6. You should be able to generate discussion about progressing your treatment and integrating and synthesising other valid treatment ideas from other team members and you will be encouraged to lead that discussion.
7. You will be expected to chair and respond to questions and take part in the clinical discussion, but the session is an opportunity to utilise the breadth of experience and thought in the room
8. You will be expected to have at least attempted to have specified treatment targets and ingredients using the RTSS format and that these will encourage discussion within the session to ground an appropriate treatment plan.

RTSS Treatment Planning

Table S2: RTSS (Rehabilitation Treatment Specifiaction System) Treatment Planning Table
Designed to encourage up to 4 treatment components (rows) to be determined. FITT – frequency / intensity / time / type [2]; LOS – length of stay; PA – physical activity

| **Targets**  (What and in what way?  How will you measure?) | **Group**  (Organ, Skill, Representation) | **Volition Type**  (Volitional ,Direct volitional, Non Volitional) | **Mechanism of Action**  (How will your treatment achieve your target) | **Ingredients**  (What will your treatment consist of?) | **Dosing & Progression**  (FITT [2] Principle) |
| --- | --- | --- | --- | --- | --- |
| **1^st^ target:** |  |  |  |  |  |
| **2^nd^ target** |  |  |  |  |  |
| **3^rd^ target** |  |  |  |  |  |
| **4^th^ target** |  |  |  |  |  |

COM-B Model


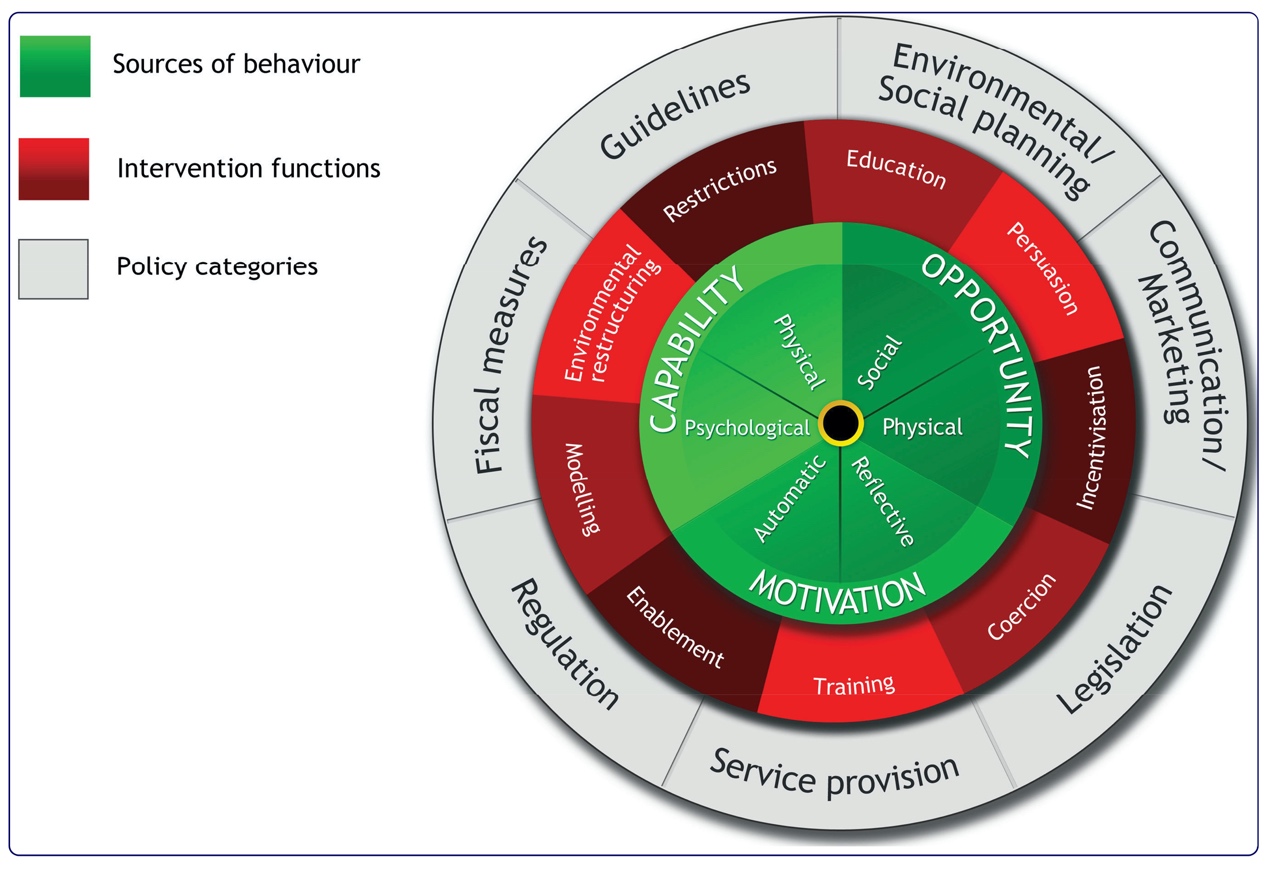


Figure S1: The Behaviour Change Wheel (Michie et al 2011 [1])
A framework used to conceptualise behaviour based on six factors. Given there is sufficient Motivation (all given automatic and/or reflective brain processes that energise and direct behaviour), then if an individual has Opportunity (all social and/or physical factors external to the individual that make the behaviour possible or prompt it;) and the Capability (the requisite psychological and/or physical knowledge and skills), then theoretically the individual has agency that allows the factors to interact without constraint to volitionally change behaviour. If there is evidence of limitation(s) in any of the behavioural factors, then the model offers intervention prompts that can be utilised depending on which behavioural factors are the target. It equally offers factors whereby policy makers might influence behavioural targets [1].

Acknowledgement

This work is done by the below authors and we are using it in our study. We have provided detailed information of the owners of this work:

Jamie Gibson^1,2^, Alexandra Curtis^1^, Gareth D. Jones^1,3^

^1^Dept. of Physiotherapy, Guy’s & St Thomas’ NHS Foundation Trust, London, UK

^2^Health Education England, Leeds, UK

^3^Centre for Human and Applied Physiological Sciences, King’s College London, UK

References

1. Michie S, van Stralen MM, West R: **The behaviour change wheel: a new method for characterising and designing behaviour change interventions**. *Implement Sci* 2011, **6**:42.

2. Liguori G, Medicine ACoS: **ACSM's guidelines for exercise testing and prescription**, 11th edn. Philadelphia, PA: Lippincott Williams & Wilkins; 2020.
