# Supplementary material for: Embedding the rehabilitation treatment specification system (RTSS) into clinical practice: an evaluation of a pilot teaching programme
Source: BMC Med Educ. 2023 Feb 2;23:85. doi: 10.1186/s12909-022-03861-2 (PMC9896736; doi:10.1186/s12909-022-03861-2)
Supplement: Supplementary file 2 — Additional file 2. Questionnaire provided to clinicians pre and post RTSS teaching programme to measure familiarity and perceived benefit of the RTSS. [file 12909_2022_3861_MOESM2_ESM.pdf]

Pre- Teaching Programme Questionnaire

* Required

1. Please state your discipline (e.g. physiotherapist) *

2. Please state your banding *

3. Please choose the answer that best matches the statement below *

	Strongly Disagree	Disagree	Neutral	Agree	Strongly Agree
I find it easy to create a problem list after assessing a patient	○	○	○	○	○
I am able to structure a treatment intervention (e.g. equipment	○	○	○	○	○

equipment, dosage, feedback, level of assistance required)

I find it difficult to interpret the treatment plan of other clinicians

I have difficulty identifying the goals of my treatment

I am confident in communicating why I have chosen a treatment intervention

Prior to a treatment session, I have difficulty planning the methods of my intervention

When viewing other therapists notes, it is clear how to replicate their treatment plans

I struggle communicating the rationale of my treatment

4. Please specify any treatment description tools you are aware of

   If you are unaware of any tools, please move to section 2

5. Please specify any treatment description tools you are currently using in your clinical practice

6. On a scale of 1-10 (10 being most beneficial) how beneficial do you find treatment description tools?

   Please explain the reasoning for your answer

7. Please include any additional comments

This content is neither created nor endorsed by Microsoft. The data you submit will be sent to the form owner.

Microsoft Forms
