# Supplementary material for: Embedding the rehabilitation treatment specification system (RTSS) into clinical practice: an evaluation of a pilot teaching programme
Source: BMC Med Educ. 2023 Feb 2;23:85. doi: 10.1186/s12909-022-03861-2 (PMC9896736; doi:10.1186/s12909-022-03861-2)
Supplement: Supplementary file 3 — Additional file 3. Outline of terminology included in frequency of controlled vocabulary audit completed at pre and post RTSS teaching Programme. [file 12909_2022_3861_MOESM3_ESM.docx]

| Adoption into clinical practice: RTSS Frequency of Controlled Vocabulary Audit | |
| --- | --- |
| Keyword | Frequency tally |
| Rehabilitation treatment specification system or RTSS |  |
| Specify/Specified/specification |  |
| Treatment component |  |
| Aim |  |
| Target |  |
| Ingredient |  |
| Mechanism of Action |  |
| Treatment group |  |
| Organ/ organ function/ organ system |  |
| Skill (including terms: Activity like or function-like) and/ or habit |  |
| Representation |  |
| Dose/dosage/dosing/dosing parameters |  |
| Progression |  |
| Volition/ Non volition |  |
