# Supplementary material for: Embedding the rehabilitation treatment specification system (RTSS) into clinical practice: an evaluation of a pilot teaching programme
Source: BMC Med Educ. 2023 Feb 2;23:85. doi: 10.1186/s12909-022-03861-2 (PMC9896736; doi:10.1186/s12909-022-03861-2)
Supplement: Supplementary file 4 — Additional file 4. Inter-rater reliability data for the Frequency of Terminology Vocabulary Audit completed at pre and post RTSS teaching Programme. [file 12909_2022_3861_MOESM4_ESM.docx]

Reliability Word Count for RTSS Controlled Vocabulary

Supplementary Material: Reliability Word Count for RTSS Controlled Vocabulary Within Clinical Case Notes

Table S1: Reliability summary of 3-raters assessing word count of controlled RTSS vocabulary across 15 paper cases Data is presented as word counts per controlled vocabulary element and as total word count. Intraclass correlation coefficients (ICCs; 2-way random effect model for absolute agreement) are shown.
The – sign represents data that was too homogenous for ICCs_(2,1)_ to be calculated; †corresponding 95%CIs with $F$ and $p$ values calculated from one-way ANOVAs

| Case No. | Rehabilitation treatment specification system or RTSS | | | Specify/Specified/ specification | | | Treatment component | | | Aim | | | Target | | | Ingredient | | | Mechanism of Action | | | Treatment group | | | Organ/ organ function/ organ system | | | Skill (including activity-like or function-like) and/or habit | | | Representation | | | Dose/dosage/dosing/ dosing parameters | | | Progression | | | Volition/ Non volition | | | TOTAL WORD COUNT | | |
| --- | --- | --- | --- | --- | --- | --- | --- | --- | --- | --- | --- | --- | --- | --- | --- | --- | --- | --- | --- | --- | --- | --- | --- | --- | --- | --- | --- | --- | --- | --- | --- | --- | --- | --- | --- | --- | --- | --- | --- | --- | --- | --- | --- | --- | --- |
|  | Rater | | | Rater | | | Rater | | | Rater | | | Rater | | | Rater | | | Rater | | | Rater | | | Rater | | | Rater | | | Rater | | | Rater | | | Rater | | | Rater | | | Rater | | |
|  | 1 | 2 | 3 | 1 | 2 | 3 | 1 | 2 | 3 | 1 | 2 | 3 | 1 | 2 | 1 | 2 | 3 | 3 | 1 | 2 | 3 | 1 | 2 | 3 | 1 | 2 | 3 | 1 | 2 | 3 | 1 | 2 | 3 | 1 | 2 | 3 | 1 | 2 | 3 | 1 | 2 | 3 | 1 | 2 | 3 |
| 1 | 1 | 1 | 1 | 1 | 1 | 3 | 3 | 2 | 1 | 0 | 0 | 0 | 3 | 3 | 3 | 2 | 1 | 2 | 0 | 0 | 2 | 0 | 0 | 0 | 0 | 0 | 0 | 3 | 3 | 3 | 2 | 2 | 2 | 5 | 5 | 5 | 0 | 0 | 0 | 0 | 1 | 1 | 20 | 19 | 23 |
| 2 | 0 | 0 | 0 | 1 | 1 | 1 | 0 | 0 | 0 | 1 | 1 | 1 | 0 | 0 | 0 | 0 | 0 | 0 | 0 | 0 | 0 | 0 | 0 | 0 | 0 | 0 | 0 | 0 | 1 | 0 | 0 | 0 | 0 | 0 | 0 | 0 | 0 | 0 | 0 | 0 | 0 | 0 | 2 | 3 | 2 |
| 3 | 0 | 0 | 0 | 0 | 0 | 0 | 0 | 0 | 0 | 0 | 0 | 1 | 0 | 1 | 1 | 0 | 1 | 0 | 0 | 0 | 0 | 0 | 0 | 0 | 0 | 1 | 1 | 0 | 2 | 2 | 0 | 1 | 1 | 0 | 8 | 4 | 0 | 1 | 2 | 0 | 0 | 0 | 0 | 15 | 12 |
| 4 | 0 | 0 | 0 | 1 | 1 | 1 | 0 | 0 | 0 | 0 | 0 | 0 | 0 | 1 | 1 | 0 | 0 | 0 | 0 | 0 | 0 | 0 | 0 | 0 | 0 | 0 | 0 | 0 | 0 | 0 | 0 | 0 | 0 | 4 | 2 | 3 | 0 | 0 | 0 | 0 | 0 | 0 | 5 | 4 | 5 |
| 5 | 0 | 0 | 1 | 1 | 2 | 1 | 0 | 0 | 0 | 0 | 0 | 0 | 2 | 2 | 3 | 0 | 0 | 0 | 0 | 0 | 0 | 0 | 0 | 0 | 0 | 0 | 0 | 2 | 2 | 2 | 0 | 0 | 0 | 1 | 0 | 3 | 2 | 2 | 3 | 0 | 0 | 0 | 8 | 8 | 13 |
| 6 | 0 | 0 | 0 | 0 | 0 | 0 | 0 | 0 | 0 | 1 | 0 | 2 | 0 | 0 | 0 | 0 | 0 | 0 | 0 | 0 | 0 | 0 | 0 | 0 | 0 | 0 | 0 | 0 | 1 | 0 | 0 | 0 | 0 | 4 | 2 | 0 | 0 | 0 | 0 | 0 | 0 | 0 | 5 | 3 | 2 |
| 7 | 0 | 0 | 0 | 0 | 0 | 0 | 0 | 0 | 0 | 0 | 0 | 0 | 0 | 0 | 0 | 0 | 0 | 0 | 0 | 0 | 0 | 0 | 0 | 0 | 0 | 0 | 0 | 0 | 0 | 0 | 0 | 0 | 0 | 3 | 1 | 0 | 0 | 0 | 0 | 0 | 0 | 0 | 3 | 1 | 0 |
| 8 | 0 | 0 | 0 | 0 | 0 | 0 | 0 | 0 | 0 | 0 | 1 | 0 | 1 | 1 | 1 | 0 | 0 | 0 | 0 | 0 | 0 | 0 | 0 | 0 | 0 | 0 | 0 | 0 | 0 | 0 | 0 | 0 | 0 | 3 | 0 | 0 | 0 | 0 | 0 | 0 | 0 | 0 | 4 | 2 | 1 |
| 9 | 0 | 0 | 0 | 0 | 1 | 1 | 0 | 0 | 0 | 0 | 0 | 0 | 0 | 0 | 0 | 1 | 1 | 1 | 0 | 0 | 0 | 0 | 0 | 0 | 0 | 0 | 0 | 0 | 0 | 0 | 0 | 0 | 0 | 4 | 2 | 1 | 0 | 1 | 1 | 1 | 1 | 1 | 6 | 6 | 5 |
| 10 | 0 | 0 | 0 | 2 | 3 | 2 | 0 | 0 | 0 | 0 | 0 | 1 | 2 | 2 | 1 | 3 | 3 | 3 | 0 | 0 | 0 | 0 | 0 | 0 | 0 | 0 | 0 | 0 | 0 | 0 | 1 | 1 | 1 | 3 | 3 | 3 | 0 | 0 | 0 | 0 | 0 | 0 | 11 | 12 | 11 |
| 11 | 0 | 0 | 0 | 1 | 0 | 1 | 0 | 0 | 0 | 1 | 0 | 1 | 0 | 1 | 1 | 1 | 1 | 1 | 0 | 0 | 0 | 0 | 0 | 0 | 0 | 0 | 0 | 1 | 0 | 2 | 0 | 0 | 0 | 0 | 0 | 0 | 0 | 0 | 0 | 0 | 0 | 0 | 4 | 2 | 6 |
| 12 | 0 | 0 | 0 | 0 | 0 | 0 | 0 | 0 | 0 | 0 | 0 | 1 | 1 | 0 | 1 | 0 | 0 | 0 | 0 | 0 | 1 | 0 | 0 | 0 | 0 | 0 | 0 | 0 | 0 | 0 | 0 | 0 | 0 | 6 | 0 | 4 | 0 | 0 | 1 | 0 | 0 | 0 | 7 | 0 | 8 |
| 13 | 0 | 0 | 0 | 0 | 0 | 0 | 0 | 0 | 0 | 0 | 0 | 0 | 2 | 2 | 2 | 2 | 2 | 2 | 0 | 0 | 0 | 0 | 0 | 0 | 0 | 0 | 0 | 1 | 4 | 1 | 0 | 0 | 0 | 3 | 1 | 1 | 0 | 0 | 0 | 1 | 1 | 1 | 9 | 10 | 7 |
| 14 | 0 | 0 | 0 | 0 | 0 | 0 | 0 | 0 | 0 | 0 | 0 | 0 | 0 | 0 | 0 | 0 | 0 | 0 | 0 | 0 | 0 | 0 | 0 | 0 | 0 | 0 | 0 | 0 | 0 | 0 | 0 | 0 | 0 | 1 | 5 | 0 | 0 | 0 | 0 | 0 | 0 | 0 | 1 | 5 | 0 |
| 15 | 0 | 0 | 0 | 0 | 0 | 0 | 0 | 0 | 0 | 0 | 1 | 0 | 1 | 1 | 1 | 2 | 2 | 2 | 0 | 0 | 0 | 0 | 0 | 0 | 0 | 0 | 0 | 2 | 3 | 2 | 0 | 0 | 0 | 4 | 4 | 5 | 0 | 0 | 0 | 0 | 0 | 0 | 9 | 11 | 10 |
| Total | 1 | 1 | 2 | 7 | 9 | 10 | 3 | 2 | 1 | 3 | 3 | 7 | 12 | 14 | 15 | 11 | 11 | 11 | 0 | 0 | 3 | 0 | 0 | 0 | 0 | 1 | 1 | 9 | 16 | 12 | 3 | 4 | 4 | 41 | 33 | 29 | 2 | 4 | 7 | 2 | 3 | 3 | 94 | 101 | 105 |
| ${ICC}_{(2,1)}$ | 0.741 | | | 0.739 | | | 0.786 | | | 0.236 | | | 0.865 | | | 0.956 | | | – | | | – | | | – | | | 0.695 | | | 0.935 | | | 0.254 | | | 0.737 | | | 0.857 | | | 0.747 | | |
| (95%CI) | (0.478 to 0.888) | | | (0.504 to 0.893) | | | (0.579 to 0.914) | | | (-0.050 to 0.582) | | | (0.718 to 0.947) | | | (0.900 to 0.984) | | | – | | | – | | | – | | | (0.441 to 0.872) | | | (0.855 to 0.975) | | | (-0.053 to 0.604) | | | (0.491 to 0.893) | | | (0.704 to 0.944) | | | (0.511 to 0.897) | | |
| †$F$(2) | 1.000 | | | 0.867 | | | 1.000 | | | 1.882 | | | 1.181 | | | 0.000 | | | – | | | – | | | – | | | 2.089 | | | 1.000 | | | 0.749 | | | 3.746 | | | 1.000 | | | 0.251 | | |
| $p$ | 0.381 | | | 0.431 | | | 0.381 | | | 0.171 | | | 0.322 | | | 1.000 | | | – | | | – | | | – | | | 0.143 | | | 0.381 | | | 0.482 | | | 0.036 | | | 0.381 | | | 0.780 | | |
