# Supplementary material for: Embedding the rehabilitation treatment specification system (RTSS) into clinical practice: an evaluation of a pilot teaching programme
Source: BMC Med Educ. 2023 Feb 2;23:85. doi: 10.1186/s12909-022-03861-2 (PMC9896736; doi:10.1186/s12909-022-03861-2)
Supplement: Supplementary file 5 — Additional file 5. Clinical Reasoning Rubric of Documentation Assessment used to measure quality of clinical reasoning in SOAP documentation, complete at pre and post RTSS teaching programme. [file 12909_2022_3861_MOESM5_ESM.docx]

| Note Assessment: Clinical Reasoning Rubric of Documentation Assessment | | | |
| --- | --- | --- | --- |
|  | Low Quality | Moderate Quality | High |
| **Problem Identification and Prioritization** | Problems lacking relevance to assessment, little to low evidence of prioritization of problems. Vague specification of problems. (e.g. weakness) | Most problems relevant to assessment. Some evidence of prioritization. Moderately specified problems (e.g. LL weakness). | All generated problems relevant from assessment. Fully prioritized problem list. Problems generated are fully specified. (e.g. low level weakness of L ankle) |
| **Treatment Goals** | Less than approx. 50% of problems have specific and appropriate therapeutic goals. | Appropriate therapeutic goals for a few identified problems. (Approx. 50%-80%). | Appropriate therapeutic goals for most identified problem (Approx. >80%). |
| **Treatment Plan** | Less than approx. 50% of problems have an appropriate and complete treatment plan.  Little to no specificity of recommendations (including treatment name, intensity, route, frequency, and duration of therapy) for each identified problem) | Partially complete and/or inappropriate for a few identified problems (approx. 50%-80%).  Moderate specificity of recommendations (including treatment name, intensity, route, frequency, and duration of therapy) for each identified problem) | Specific, appropriate and justified (matches problem list and treatment goals)  Full specificity of recommendations for each identified problem (including treatment name, intensity, route, frequency, and duration of therapy) for each identified problem) |
