# Supplementary material for: Embedding the rehabilitation treatment specification system (RTSS) into clinical practice: an evaluation of a pilot teaching programme
Source: BMC Med Educ. 2023 Feb 2;23:85. doi: 10.1186/s12909-022-03861-2 (PMC9896736; doi:10.1186/s12909-022-03861-2)
Supplement: Supplementary file 6 — Additional file 6. Script that guided semi structured focus group used to measure adoption and reflection of the RTSS teaching Programme. [file 12909_2022_3861_MOESM6_ESM.docx]

Focus Group: RTSS Pilot teaching programme:

Aim

- Through understanding the efficacy of the teaching programme underpinning embedding the RTSS into clinical practice, to develop the programme so it best meets the needs of staff

Objectives

- To understand the pilot groups’ thoughts around the teaching design that was implemented, specifically;
  - Mixed model of lectures and problem based learning
  - Frequency, intensity and duration of content delivery
  - Supportive teaching resources
- To explore pilot groups understanding of the RTSS following the teaching program
- To understand the facilitators and barriers to the pilot group applying the RTSS to their clinical practice

Methodology:

Setup:

- Environment: Booked quiet room, circular setup of chairs (with round table preferably).
- Seating plan: quieter individuals opposite moderator, more talkative next to moderator.
- Dictaphone for recording
- Timer for referencing written and audio recordings
- 1 lead moderator, 1 assistant moderator

Participants:

- Recruitment via contacting senior members of the Inpatient Oncology Physiotherapy team
- Staff from the inpatient oncology team
- Staff who attended the majority of sessions (4/6 sessions)
- 4-6 members participants (depending on staffing availability
- 1 staff member representing each staff banding 3-8 (depending on staffing availability)

Pre discussion/Introduction:

- Arrival of moderators 15 minutes prior to session to setup environment and test equipment.
- Assignment of participant number, seated as per seating plan above.
- Introduction message “As you know, you were the selected group to pilot the initial RTSS teaching program, the aim of this program was to teach clinicians an understanding of the RTSS that could be applied to clinical practice.

With this focus group; ideally, we'd just like you all to talk to each other about how you found the teaching program and the RTSS itself. To try and get your views of what's important and not ours, we want to take as little part in this as possible; should also emphasize that we're not testing you, to see if you give ‘right’ or ‘wrong’ answers. We have some questions we can ask to get the ball rolling or steer things back on course if the discussion goes too far off track. You can say as much or as little as you want; we might try and come in on the conversation if it seems that someone isn't getting a chance to chip in.

The conversation's being tape-recorded for us to transcribe and look at later. One of us will be taking notes; this is just to make it easier to know who's talking when we're listening back. However, it's only us that will ever hear the tape, and nobody will be named on the transcript.

We'll use the information today to guide future delivery of the program to other therapists and we will be more than happy to let you know the outcomes of this.

Do you have any questions? You can always get back to us later

Discussion Overview

| Introduction and background | 5mins |
| --- | --- |
| - Following the 6 sessions, how do you feel about your understanding of the RTSS?   - Is there anything you would change to future sessions to improve understanding?   - How would this have helped your understanding? - How did you find the mixed teaching approach of lectures and applied learning to case studies?   - Why did this approach work/not work for you?   - Would you have changed anything?   - If we had made the change suggested, how would that have helped you? - What do you think are the benefits of using the RTSS in clinical practice? - What barriers are there to using the RTSS in clinical practice?   - Is there anything that we could cover in the sessions that would help you overcome this barrier? - Following the teaching, what changes have you noticed in your treatment planning?   - If no change, probe as to why? - In relation to the RTSS, what questions remain unanswered from the sessions for you? - As you know, we are going to be implementing a teaching programme to other therapists, based on your experiences and our discussion today, what can we do to improve the teaching program? - Is there anything else that anyone feels that we should’ve covered, but didn’t? | 50 mins |
| Thank and close | 5mins |
| **Total time** | **60mins** |
